# Differential leukocyte counts and cardiovascular mortality in very old patients with acute myocardial infarction: a Chinese cohort study

**DOI:** 10.1186/s12872-020-01743-3

**Published:** 2020-10-28

**Authors:** Xiao-Ni Yan, Jing-Lu Jin, Meng Zhang, Li-Feng Hong, Yuan-Lin Guo, Na-Qiong Wu, Cheng-Gang Zhu, Qian Dong, Jian-Jun Li

**Affiliations:** 1 State Key Laboratory of Cardiovascular Disease, Fu Wai Hospital, National Center for Cardiovascular Diseases, Chinese Academy of Medical Sciences, Peking Union Medical College, BeiLiShi Road 167, Beijing, 100037 China; 2 Division of Cardiology, The Fifth Hospital of Wuhan & Cardiovascular Insititute of Jianghan University, Wuhan, 430050 China

**Keywords:** Leukocyte, Mortality, Acute myocardial infarction, Very old patients

## Abstract

**Background:**

Total leukocyte and differential Leukocyte counts are prognostic indictors in patients with coronary artery disease (CAD). However, there is no data available regarding their prognostic utility in very old patients with acute myocardial infarction (AMI). The aim of this study is to investigate the potential role of different leukocyte parameters in predicting the mortality among very old patients with AMI.

**Methods:**

A total of 523 patients aged over 80 years with AMI were consecutively enrolled into this study. Leukocyte and its subtypes were obtained at admission in each patient. The primary study endpoint was cardiovascular mortality. Patients were followed up for an average of 2.2 years and 153 patients died. The associations of leukocyte parameters with mortality were assessed using Cox regression analyses. The concordance index was calculated to test the model efficiency.

**Results:**

In multivariable regression analysis, neutrophils-plus-monocytes-to-lymphocytes ratio (NMLR) and neutrophils-to-lymphocytes ratio (NLR) were two most significant predictors of mortality among all the leukocyte parameters (HR = 3.21, 95% CI 1.75–5.35; HR = 2.79, 95% CI 1.59–4.88, respectively, all *p *< 0.001, adjusted for age, male gender, body mass index, family history of CAD, smoking, hypertension, diabetes mellitus, high-density lipoprotein cholesterol (HDL-C), non-HDL-C, high sensitivity C-reactive protein, creatinine, left ventricular ejection fraction, troponin I, use of statin, angiotensin-converting enzyme inhibitors/angiotensin receptor blockers, and percutaneous coronary intervention). Furthermore, adding NMLR and NLR into the Cox model increased the C-statistic by 0.038 and 0.037 respectively, which were more significant than that of other leukocyte parameters. Besides, addition of NMLR and NLR to the Canada Acute Coronary Syndrome Risk Score model also increased the C-statistic by 0.079 and 0.077 respectively.

**Conclusion:**

Our data firstly indicated that most leukocyte subtypes were independent markers for the mortality in very old patients with AMI, while NMLR and NLR appeared to be more effective.

## Background

Acute myocardial infarction (AMI) is a major cause of mortality worldwide, especially in geriatric patients [1]. Previous studies suggested that elderly patients with AMI had higher in-hospital, short- and long-term mortality rates compared with that of the young adults [2–5]. Moreover, the prevalence of AMI was much higher in very old patients [6]. Thus, finding new markers for precise cardiovascular risk stratification in very old patients may be crucial. In fact, several biomarkers including C-reactive protein and brain natriuretic peptide were proved to be predictive of adverse outcomes in AMI patients [7]. However, measurements of these markers are costly in current clinical practice. It may be of great value to find a simple and easy to access biomarker for predicting mortality in very old patients with AMI.

It was previously demonstrated that leukocyte count was a simple and low-cost marker which had a key role in the pathophysiology and prognosis of cardiovascular disease. Previous studies showed that leukocyte count was associated with a higher risk of coronary artery disease (CAD) [8–10]. Moreover, leukocyte count was also related to the severity of coronary artery stenosis, as well as the extent of infarct size in patients with AMI [11–13]. Furthermore, elevated leukocyte count was correlated with higher short- and long-term mortality in stable CAD patients with previous MI, unstable angina pectoris or acute coronary syndrome (ACS) [14–18].

As it is well known, the leukocytes subpopulations have different impacts in the development of atherosclerosis. Several studies had indicated that leukocytes and its subtypes were related to mortality in patients with ACS. However, the results were controversial [19, 20]. Previous studies about prognostic value of leukocytes were with several limitations as heterogeneous study population and limited subtypes of leukocyte. More importantly, to the best of our knowledge, data about leukocyte and its subtypes predicting mortality of AMI in very old patients with AMI is currently unavailable. Besides, no study investigated the relationship between neutrophil-plus-monocyte-to-lymphocyte ratio (NMLR) and clinical outcome in very old patients with AMI. In the current study, we aim to assess the association between leukocyte and its subpopulations with mortality and identify which leukocyte subtype is the best marker for predicting mortality in very old patients with AMI.

## Methods

### Study population

The study was complied with the Declaration of Helsinki and was approved by the hospital ethical review board (FuWai Hospital and National Center for Cardiovascular Diseases, Beijing, China). All patients provided written informed consent before enrolling in our study.

**Fig. 1:**
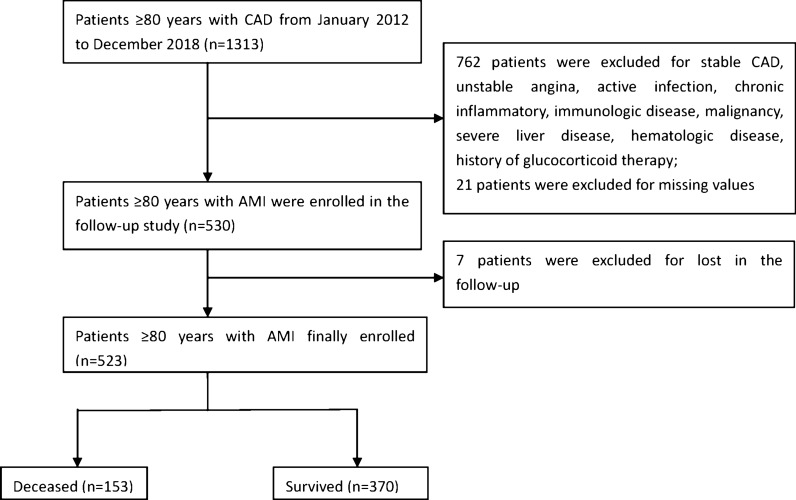
Flow chart of the study design. *CAD* coronary artery disease, *AMI* acute myocardial infarction

As described in Fig. 1, From January 2012 to December 2018, we consecutively enrolled 1313 hospitalized patients ≥ 80 years with CAD at our center. Patients were excluded from our study for the following criteria: stable CAD, unstable angina (UA), active infection, chronic inflammatory, immunologic disease, malignancy, severe liver disease, chronic obstructive pulmonary disease, hematologic disease, history of glucocorticoid therapy within the past 3 months. Finally, a total of 523 patients over 80 years with AMI were included in the present study. The medical history was recorded, and the electrocardiogram, clinical, laboratory examinations of each patient were carried out by experienced nurses and physicians.

The diagnosis of AMI was made by clinical presentation, electrocardiography and increment in cardiac troponins. Dyslipidemia was defined in subjects with fasting total cholesterol (TC) concentration ≥ 200 mg/dL, and triglyceride (TG) concentration ≥ 150 mg/dL. Diabetes mellitus (DM) was diagnosed as fasting plasma glucose (FPG) ≥ 7.0 mmol/L, the haemoglobin A1c (HbA1c) ≥ 6.5% or the current use of insulin or oral hypoglycemic drugs. Hypertension was diagnosed if a patient had blood pressure ≥ 140/90 mmHg three or more consecutive times or had been receiving anti-hypertensive medications.

### Laboratory tests

Venous blood samples were drawn from patients at admission. We measured the level of white blood cells, neutrophils, monocytes and lymphocytes using an automated hematology analyzer Counter LH780 (Beckman Coulter Ireland Inc, Mervue, Galway, Ireland). Neutrophil-to-lymphocyte ratio (NLR), monocyte-to-lymphocyte ratio (MLR), eosinophil-to-leukocyte ratio (ELR) and NMLR were calculated. Lipid biomarkers including plasma TC, low-density lipoprotein cholesterol (LDL-C), high-density lipoprotein cholesterol (HDL-C), TG were assayed by automatic biochemistry analyser (Hitachi 7150, Tokyo, Japan). The level of non-HDL-C was calculated from the TC subtracted by HDL-C. The level of high sensitivity C-reactive protein (hsCRP) was determined using immunoturbidimetry (Beckmann Assay 360; Bera, CA, USA). Cardiac troponin I (cTnI) was measured by an immunochemiluminometric assay (Access AccuTnI, Beckman Coulter, CA). The rest of biochemical parameters were analyzed with standard laboratory techniques. The left ventricular ejection fraction (LVEF), which was evaluated by experienced specialists who performed the transthoracic echocardiography in a standard manner, was calculated using either Teichholz or Simpson’s biplane method at admission. The Canada Acute Coronary Syndrome Risk Score (C-ACS), included 4 variables and ranged from 0 to 4, was calculated as follows: 1 point was given for age ≥ 75 years, Killip > 1, systolic blood pressure < 100 mmHg, and heart rate > 100 beats/min, respectively [21].

### Study endpoints

Follow-up data were acquired by interviewing each patient face to face or by telephone. The primary endpoint used for the analysis was cardiovascular mortality mainly including death caused by AMI, congestive heart failure, stroke, malignant arrhythmia and other structural or functional cardiac diseases as our previous studies [22]. Mortality data were obtained from interviewing and medical records.

### Statistical analysis

Research data were analyzed using SPSS version 22.0 software (Chicago, IL, USA) and R language version 3.6.0 (Feather Spray). The Kolmogorov–Smirnov test was used to test the normality distribution. Data were shown as mean ± standard deviations (SD) or median with interquartile range for continuous variables and as numbers and percentages for categorical variables. Comparisons were made using Student’s *t* test, Mann–Whitney *U* test, Kruskal–Wallis test, chi-square test, or Fisher’s exact test when appropriate. Kaplan–Meier analysis was performed to illustrate mortality differences by tertiles of NMLR and NLR and Log-rank test was carried out to assess significance. Univariable and multivariable Cox regression analyses were used to calculate the hazard ratios (HRs). The adjusted Cox models included traditional cardiovascular risk factors as follows: age, male gender, body mass index (BMI), family history of CAD, smoking, hypertension, DM, HDL-C, non-HDL-C, hsCRP, creatinine, LVEF, troponin I, percutaneous coronary intervention (PCI), use of statin and angiotensin-converting enzyme inhibitors/angiotensin receptor blockers (ACE-I/ARB). Besides, the predictive values of original model, C-ACS model, and either original model or C-ACS model plus leukocyte subtypes on mortality were also analyzed by receiver-operating characteristic curve (ROC). Moreover, the concordance index (C-Statistic) was calculated to assess the predictive efficiency. The incremental values of adding leukocyte and its subtypes to traditional risk model and C-ACS model were evaluated by △C-statistic. A *p* value of less than 0.05 was considered statistically significant.

## Results

### Baseline characteristics between deceased and survived group

The study cohort consisted of 523 patients with AMI. The mean age of study population was 82.75 ± 2.74 years (median = 82 years; ranged from 80 to 93 years). The median duration of follow up was 803 days (interquartile range 323–1158 days). 153/523 (29%) died over the course of the study.

The clinical characteristics and laboratory data of all patients were shown in Table 1. In brief, the deceased group had higher mean age, hsCRP, creatinine, prevalence of DM, but lower LVEF. In addition, the deceased group had significantly higher leukocytes, neutrophils, monocytes, NMLR, NLR, MLR levels but lower lymphocytes, eosinophils, basophils, ELR when compared with the survived group (all *p* < 0.05).

**Table 1:** Baseline characteristics of studied patients

Variables	Overall (n = 523)	Deceased (n = 153)	Survived (n = 370)	*p* value
*Clinical characteristics*				
Age, years	82.75 ± 2.74	83.32 ± 3.11	82.55 ± 2.55	0.006
Male, n (%)	329(60)	109(62)	220(59)	0.576
BMI, kg/m^2^	23.77 ± 3.50	23.28 ± 3.84	23.96 ± 3.34	0.078
Smoking, n (%)	215(40)	71 (43)	141 (39)	0.445
Family history of CAD, n (%)	55(10)	22 (13)	33 (9)	0.307
Hypertension, n (%)	409(75)	134(76)	275 (74)	0.671
Diabetes mellitus, n (%)	221(41)	85 (49)	136(37)	0.005
*MI type*				
STEMI	244(47)	64(42)	180(49)	0.211
NSTEMI	279(53)	89(58)	190(51)	
*Laboratory test*				
Triglyceride, mmol/L	1.22 (0.93–1.56)	1.21 (0.92–1.59)	1.23 (0.93–1.52)	0.887
TC, mmol/L	3.81 ± 0.94	3.78 ± 0.93	3.82 ± 0.94	0.623
HDL-C, mmol/L	1.09 ± 0.29	1.05 ± 0.29	1.11 ± 0.29	0.052
LDL-C, mmol/L	2.24 ± 0.78	2.20 ± 0.78	2.26 ± 0.77	0.356
Lipoprotein(a), mg/L	203.69 (95.98–438.00)	220.44 (101.04–409.51)	202.00 (93.92–460.02)	0.552
Creatinine, μmol/L	106.86 ± 40.54	122.56 ± 50.36	100.38 ± 33.71	< 0.001
hsCRP, mg/L	4.88 (1.84–11.28)	8.29 (3.06–12.20)	4.19 (1.56–10.68)	< 0.001
LVEF (%)	52.73 ± 10.73	49.47 ± 11.86	54.29 ± 9.79	< 0.001
CK-MB, ng/mL	27.65 ± 58.10	30.94 ± 62.07	26.26 ± 56.37	0.421
Troponin I, ng/mL	4.86 ± 8.64	3.73 ± 6.65	7.58 ± 11.73	< 0.001
*Leukocyte parameters*				
Leukocytes, 10^9^/L	7.42 ± 2.81	8.21 ± 4.43	7.13 ± 2.41	< 0.001
Neutrophils, 10^9^/L	5.28 ± 2.67	6.09 ± 3.58	4.91 ± 2.09	< 0.001
Lymphocytes, 10^9^/L	1.46 ± 0.60	1.28 ± 0.57	1.53 ± 0.61	< 0.001
Monocytes, 10^9^/L	0.48 ± 0.20	0.52 ± 0.22	0.47 ± 0.18	0.013
Eosinophils, 10^9^/L	0.15 ± 0.17	0.12 ± 0.14	0.16 ± 0.19	0.008
Basophils, 10^9^/L	0.02 ± 0.02	0.02 ± 0.02	0.02 ± 0.03	< 0.001
Neutrophil-to-lymphocyte ratio	4.58 ± 4.57	6.21 ± 6.72	3.83 ± 2.86	< 0.001
Monocyte-to-lymphocyte ratio	0.38 ± 0.22	0.48 ± 0.30	0.34 ± 0.17	< 0.001
NMLR	4.96 ± 4.73	7.15 ± 7.13	4.17 ± 2.96	< 0.001
Eosinophil-to-leukocyte ratio	0.02 ± 0.03	0.02 ± 0.02	0.02 ± 0.03	0.005
*Treatment at admission*				
Aspirin, n (%)	504(96.2)	147 (95.5)	357(96.5)	0.618
Statin, n (%)	486(92.7)	137(89.0)	349 (94.3)	0.041
Beta-blocker, n (%)	479(91.4)	136(88.3)	343(92.7)	0.102
CCB, n (%)	114(21.8)	30(19.5)	84(22.7)	0.486
ACE-I/ARB, n (%)	279(53.3)	69 (45.1)	210(56.8)	0.016
Clopidogrel, n (%)	486(92.7)	142 (92.2)	344(93.0)	0.716
Ticagrelor, n (%)	32(6.3)	10(6.5)	22(5.9)	0.842
CABG	5(1.0)	1(0.6)	4(1.1)	1.000
PCI	228(43.5)	38(24.7)	190(51.4)	< 0.001

Data were expressed as mean ± SD, median (Q1–Q3 quartiles) or n (%)

*BMI* body mass index, *CAD* coronary artery disease, *MI* myocardial infarction, *STEMI* ST-segment elevation myocardial infarction, *NSTEMI* Non-ST-segment elevation myocardial infarction, *TC* total cholesterol, *LDL-C* low-density lipoprotein cholesterol, *HDL-C* high-density lipoprotein cholesterol, *hsCRP* high sensitivity C-reactive protein, *LVEF* left ventricular ejection fraction, *NMLR* neutrophil-plus-monocyte-to-lymphocyte ratio, *CCB* calcium channel blockers, *ACE-I* angiotensin-converting enzyme inhibitors, *ARB* angiotensin receptor blockers, *CABG* coronary artery bypass grafting, *PCI* percutaneous coronary intervention, *SD* standard deviation

### Predictive value of leukocyte and its subtypes for mortality

**Fig. 2:**
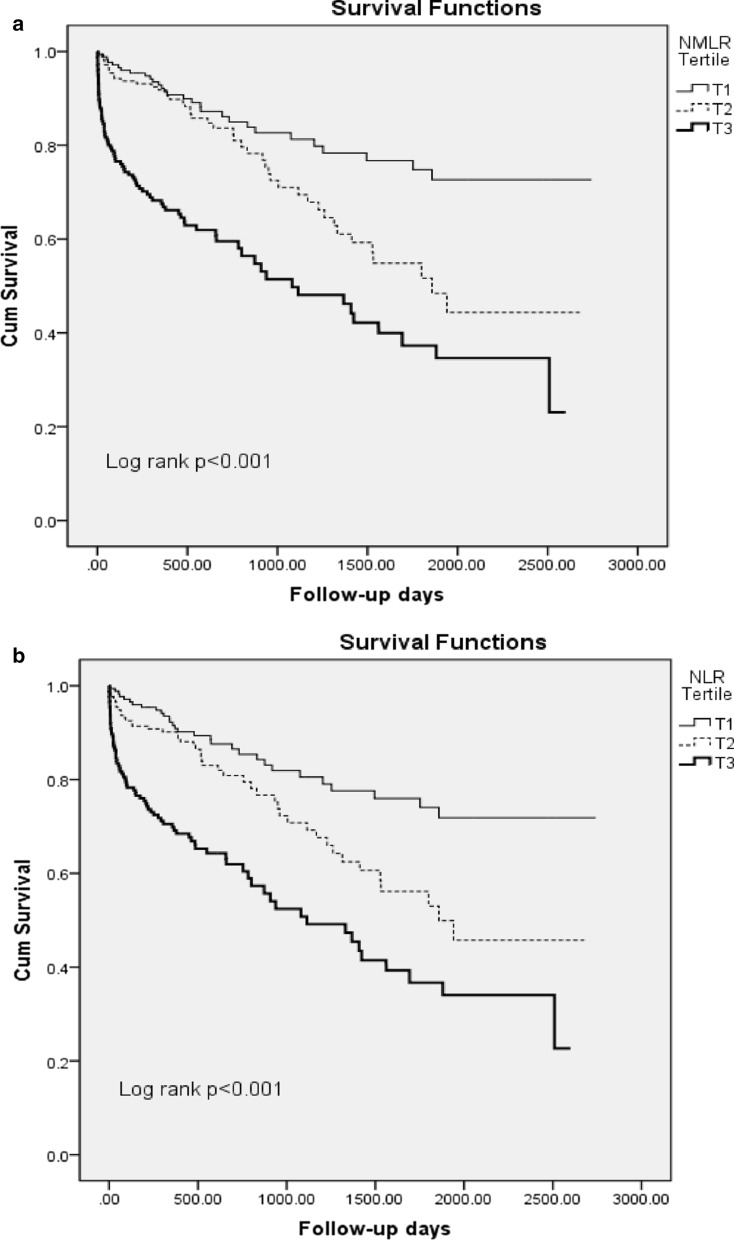
Kaplan–Meier curve for long-term survival according to tertiles of NMLR (**a**) and NLR (**b**). *NMLR* neutrophil plus monocyte to lymphocyte ratio, *NLR* neutrophil to lymphocyte ratio

As shown in Fig. 2, Kaplan–Meier analyses suggested that subjects in the T2 and T3 of NMLR and NLR had lower survival rate than that in the T1 of NMLR and NLR. As was shown in Additional file 1: Table S1, patients with older age, DM, elevated hsCRP, creatinine and Troponin I or decreased LVEF and BMI were with higher risk of cardiovascular mortality. The relationship of leukocyte and its subtypes to the cardiovascular mortality of AMI was also evaluated using Cox proportional hazard regression analyses. In univariate models, both NMLR and NLR T3 were associated with higher risk of cardiovascular mortality [NMLR: Hazard Ratio (HR): 4.05, 95% CI 2.64–6.22; NLR: HR: 3.74, 95% CI 2.44–5.71, all *p* < 0.05, Table 2]. Adjustment of confounding variables did not change the association (HR = 3.21, 95% CI 1.75–5.35; and HR = 2.79, 95% CI 1.59–4.88, respectively, all *p* < 0.001).

**Table 2:** Unadjusted and adjusted hazard ratios and 95% confidence intervals for cardiovascular mortality according to the tertitles of leukocyte and its subtypes

Variables	Tertile 1	Tertile 2	Tertile 3	*P* value
*Leukocytes*				
Unadjusted	1.00	1.02 (0.67–1.55)	1.72 (1.16–2.53)	0.005
Adjusted^a^	1.00	0.79 (0.47–1.31)	1.14 (0.66–1.96)	0.288
*Neutrophils*				
Unadjusted	1.00	1.93 (1.24–3.00)	2.86(1.87–4.38)	< 0.001
Adjusted^a^	1.00	2.02 (1.15–3.53)	2.53(1.40–4.55)	0.008
*Lymphocytes*				
Unadjusted	1.00	0.53 (0.37–0.77)	0.38(0.26–0.57)	< 0.001
Adjusted^a^	1.00	0.51(0.33–0.81)	0.47(0.28–0.77)	0.002
*Monocytes*				
Unadjusted	1.00	1.19(0.78–1.80)	1.49(1.00–2.23)	0.132
Adjusted^a^	1.00	1.15(0.70–1.88)	0.97(0.58–1.63)	0.769
*Eosinophils*				
Unadjusted	1.00	0.40(0.27–0.58)	0.42(0.29–0.63)	< 0.001
Adjusted^a^	1.00	0.46(0.29–0.73)	0.46(0.28–0.76)	0.001
*Basophils*				
Unadjusted	1.00	0.36(0.23–0.55)	0.31(0.18–0.55)	< 0.001
Adjusted^a^	1.00	0.37(0.21–0.66)	0.28(0.13–0.61)	0.001
*NMLR*				
Unadjusted	1.00	1.78 (1.11–2.84)	4.05 (2.64–6.22)	< 0.001
Adjusted^a^	1.00	1.31 (0.75–2.31)	3.21(1.75–5.35)	< 0.001
*NLR*				
Unadjusted	1.00	1.81(1.14–2.87)	3.74(2.44–5.71)	< 0.001
Adjusted^a^	1.00	1.33(0.76–2.34)	2.79(1.59–4.88)	< 0.001
*MLR*				
Unadjusted	1.00	1.53(0.96–2.44)	3.30(2.17–5.02)	< 0.001
Adjusted^a^	1.00	1.41(0.82–2.44)	2.11(1.22–3.67)	0.024
*ELR*				
Unadjusted	1.00	0.41(0.28–0.60)	0.40(0.27–0.59)	< 0.001
Adjusted^a^	1.00	0.38 (0.23–0.61)	0.46(0.28–0.75)	< 0.001

^a^Adjusted for age, male gender, BMI, Family history of CAD, smoking, hypertension, DM, HDL-C, non-HDL-C, hsCRP, Creatinine, LVEF, Troponin I, use of statin, ACE-I/ARB, PCI

*BMI* body mass index, *CAD* coronary artery disease, *DM* diabetes mellitus, *HDL-C* high-density lipoprotein cholesterol, *PCI* percutaneous coronary intervention, *hsCRP* high sensitivity C-reactive protein, *NMLR* neutrophil-plus-monocyte-to-lymphocyte ratio, *NLR* neutrophil-to-lymphocyte ratio, *MLR* monocyte-to-lymphocyte ratio, *ELR* eosinophil-to-leukocyte ratio, *ACE-I* agiotensin-converting enzyme inhibitors, *ARB* angiotensin receptor blockers

**Fig. 3:**
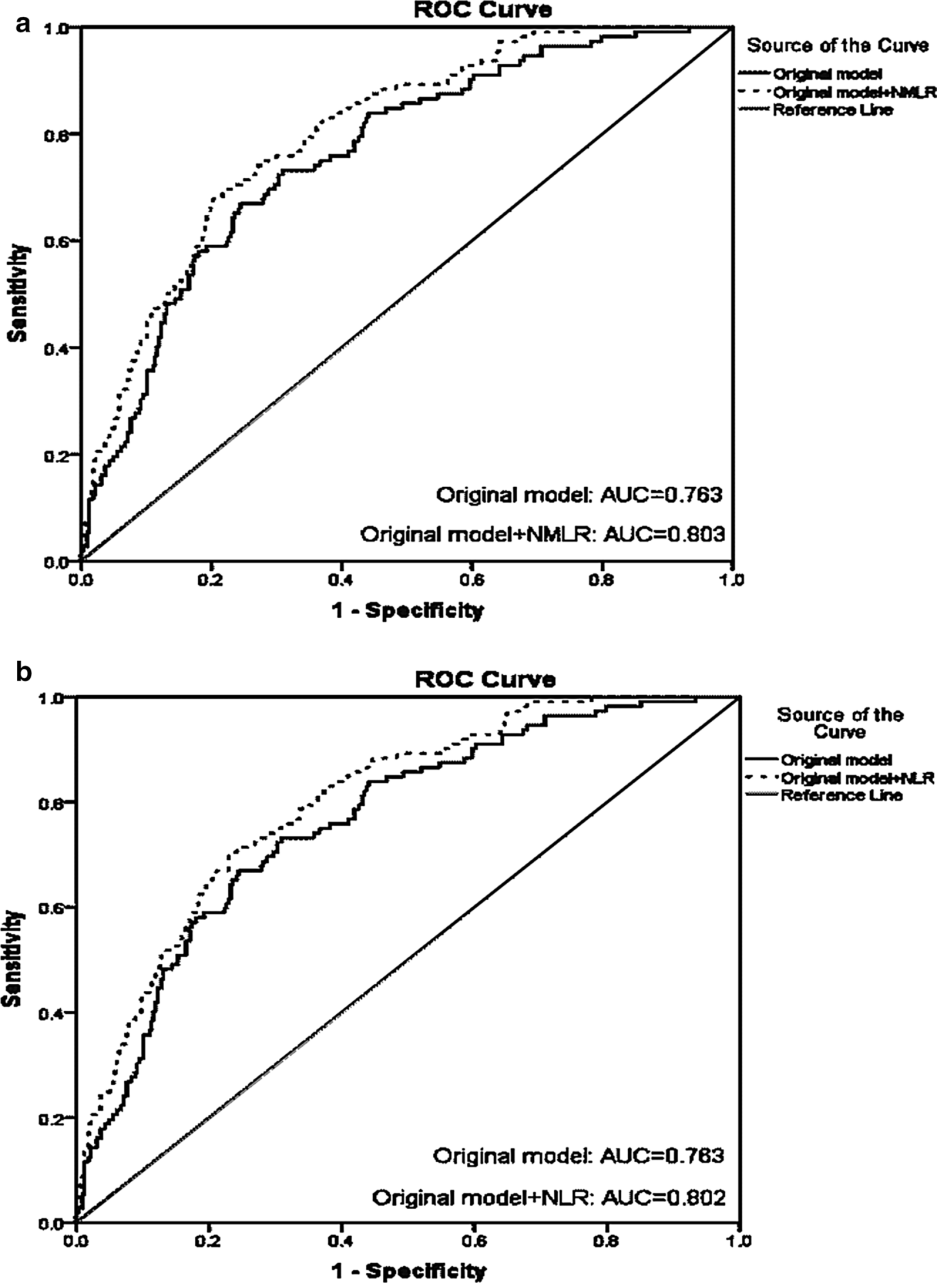
ROC curve analyses of original model, NMLR plus original model, NLR plus original model for predicting cardiovascular mortality. *ROC* receiver operating characteristic, *NMLR* neutrophil-plus-monocyte-to-lymphocyte ratio, *NLR* neutrophil-to-lymphocyte ratio

**Fig. 4:**
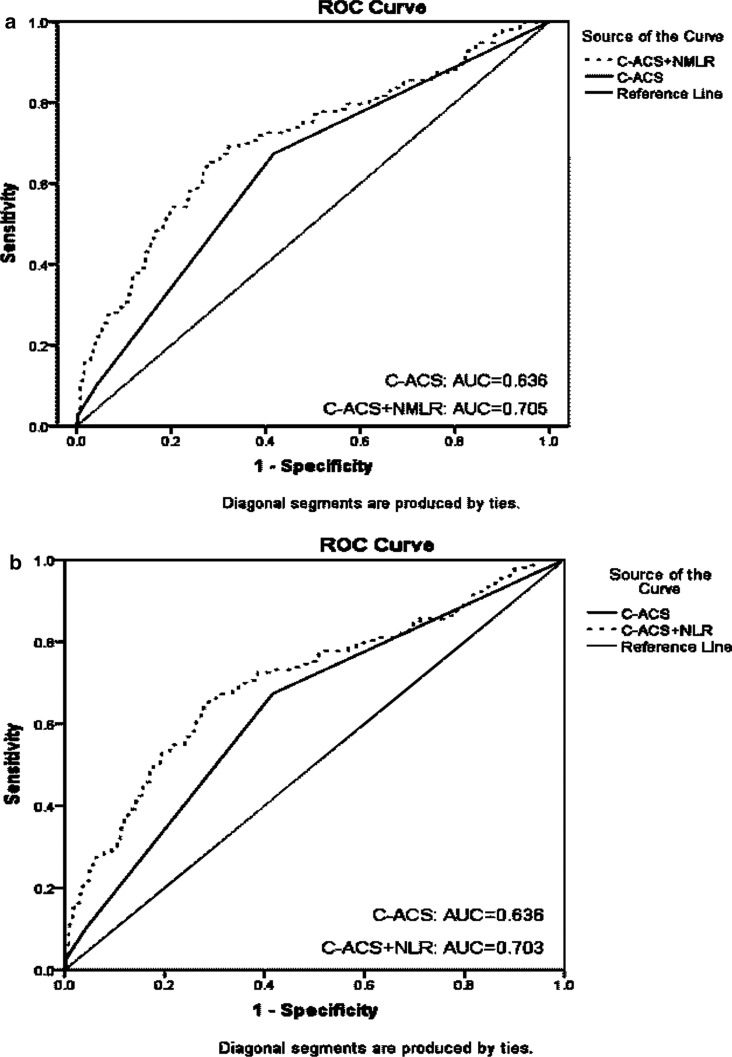
ROC curve analyses of C-ACS model, NMLR plus C-ACS model, NLR plus C-ACS model for predicting cardiovascular mortality. *ROC* receiver operating characteristic, *C-ACS* Canada Acute Coronary Syndrome Risk Score, *NMLR* neutrophil-plus-monocyte-to-lymphocyte ratio, *NLR* neutrophil-to-lymphocyte ratio

The ROC analyses of adding NMLR and NLR into C-ACS model and original model (included age, male gender, BMI, family history of CAD, smoking, hypertension, DM, HDL-C, non-HDL-C, hsCRP, creatinine, LVEF, troponin I, use of statin, ACE-I/ARB, and PCI) were shown in Figs. 3 and 4 and Table 3. The C-ACS model and original model were with the AUC of 0.636 (95% CI 0.583–0.688, *p* < 0.001) and 0.763 (95% CI 0.714–0.812, *p* < 0.001). Moreover, C-ACS model plus NMLR and NLR had the AUC of 0.705 (95% CI 0.654–0.756) and 0.703 (95% CI 0.651–0.754) respectively. Original model plus NMLR and NLR had the AUC of 0.803 (95% CI 0.759–0.847) and 0.802 (95% CI 0.757–0.846) respectively.

**Table 3:** ROC analyses revealing the value of leukocyte and its subtypes in predicting cardiovascular mortality

Variable	AUC (95% CI)	Cut-off	Sensitivity	Specificity
Leukocytes	0.598 (0.543–0.652)	8.235	0.405	0.757
Neutrophils	0.636 (0.583–0.688)	4.135	0.778	0.430
Lymphocytes	0.373 (0.319–0.427)	1.385	0.559	0.660
Monocytes	0.570 (0.516–0.625)	0.445	0.614	0.519
Eosinophils	0.403 (0.346–0.459)	0.045	0.807	0.386
Basophils	0.355 (0.302–0.408)	0.015	0.726	0.480
NMLR	0.677 (0.626–0.728)	4.133	0.588	0.692
NLR	0.673 (0.622–0.724)	3.704	0.614	0.665
MLR	0.666 (0.614–0.718)	0.361	0.627	0.678
ELR	0.385 (0.330–0.441)	0.007	0.780	0.431
Leukocytes model	0.767 (0.718–0.816)	–	–	–
Neutrophils model	0.775 (0.726–0.823)	–	–	–
Lymphocytes model	0.784 (0.739–0.830)	–	–	–
Monocytes model	0.763 (0.714–0.813)	–	–	–
Eosinophils model	0.771 (0.721–0.820)	–	–	–
Basophils model	0.792 (0.743–0.841)	–	–	–
NMLR model	0.803 (0.759–0.847)	–	–	–
NLR model	0.802 (0.757–0.846)	–	–	–
MLR model	0.788 (0.742–0.834)	–	–	–
ELR model	0.772 (0.722–0.822)	–	–	–

Other variables in the model: age, male gender, BMI, Family history of CAD, smoking, hypertension, DM, HDL-C, non-HDL-C, hsCRP, Creatinine, LVEF, Troponin I, use of statin, ACE-I/ARB, PCI

*AUC* area under curve, *BMI* body mass index, *CAD* coronary artery disease, *DM* diabetes mellitus, *HDL-C* high-density lipoprotein cholesterol, *PCI* percutaneous coronary intervention, *hsCRP* high sensitivity C-reactive protein, *NMLR* neutrophil-plus-monocyte-to-lymphocyte ratio, *NLR* neutrophil-to-lymphocyte ratio, *MLR* monocyte-to-lymphocyte ratio, *ELR* eosinophil-to-leukocyte ratio, *ACE-I*: agiotensin-converting enzyme inhibitors, *ARB* angiotensin receptor blockers

The original model was with the C-statistic of 0.728. Among all the leukocyte parameters, adding NMLR and NLR to the original model increased the C-statistic (0.766 and 0.765, △C-statistic 0.038 and 0.037 respectively, all *p* < 0.05, Table 4). As shown in Table 5, the C-statistic value of C-ACS model was 0.643. Addition of NMLR and NLR to the C-ACS model significantly improved the C-statistic to 0.722 and 0.720, respectively.

**Table 4:** C-statistics of the different models for predicting cardiovascular mortality

Models	C-statistics (95% CI)	△C-statistics (95% CI)	*p* value
Original model	0.728 (0.681–0.775)	–	–
Original model + leukocytes	0.744 (0.697–0.791)	0.016 (0.003–0.037)	0.069
Original model + neutrophils	0.753 (0.707–0.800)	0.025 (0.024–0.046)	0.022
Original model + lymphocytes	0.740 (0.693–0.787)	0.012 (− 0.003 to 0.026)	0.106
Original model + monocytes	0.729 (0.682–0.776)	0.001 (− 0.004 to 0.006)	0.846
Original model + eosinophils	0.738 (0.689–0.789)	0.010 (− 0.005 to 0.024)	0.169
Original model + basophils	0.738 (0.690–0.787)	0.010(− 0.008 to 0.028)	0.271
Original model + NMLR	0.766 (0.721–0.810)	0.038 (0.020–0.069)	0.002
Original model + NLR	0.765 (0.720–0.809)	0.037 (0.021–0.068)	0.002
Original model + MLR	0.751 (0.708–0.794)	0.023 (0.012–0.043)	0.004
Original model + ELR	0.739 (0.690–0.789)	0.011 (− 0.007 to 0.027)	0.217

Original model included age, male gender, BMI, family history of CAD, smoking, hypertension, DM, HDL-C, non-HDL-C, hsCRP, creatinine, LVEF, troponin I, use of statin, ACE-I/ARB, PCI

△C-statistics indicated the difference in C-statistic between original model and models additionally including leukocytes, neutrophils, lymphocytes, monocytes, eosinophils, basophils, NMLR, NLR, MLR, or ELR

*BMI* body mass index, *CAD* coronary artery disease, *DM* diabetes mellitus, *HDL-C* high-density lipoprotein cholesterol, *PCI* percutaneous coronary intervention, *hsCRP* high sensitivity C-reactive protein, *NMLR* neutrophil-plus-monocyte-to-lymphocyte ratio, *NLR* neutrophil-to-lymphocyte ratio, *MLR* monocyte-to-lymphocyte ratio, *ELR* eosinophil-to-leukocyte ratio

**Table 5:** C-statistics of leukocyte subtypes and C-ACS for predicting cardiovascular mortality

Models	C-statistics (95% CI)	△C-statistics (95% CI)	*P* value
C-ACS	0.643 (0.600–0.685)	–	–
C-ACS + leukocytes	0.676 (0.626–0.726)	0.033 (0.012–0.055)	0.003
C-ACS + neutrophils	0.695 (0.646–0.744)	0.052 (0.026–0.078)	< 0.001
C-ACS + lymphocytes	0.688 (0.640–0.736)	0.046 (− 0.005 to 0.085)	0.045
C-ACS + monocytes	0.663 (0.615–0.711)	0.020 (0.002–0.039)	0.029
C-ACS + eosinophils	0.686 (0.640–0.733)	0.044(0.021–0.070)	< 0.001
C-ACS + basophils	0.670 (0.623–0.718)	0.028(0.004–0.053)	< 0.026
C-ACS + NMLR	0.722 (0.675–0.768)	0.079 (0.042–0.115)	< 0.001
C-ACS + NLR	0.720 (0.674–0.767)	0.077 (0.041–0.112)	< 0.001
C-ACS + MLR	0.712 (0.669–0.754)	0.069 (0.027–0.107)	< 0.001
C-ACS + ELR	0.686 (0.639–0.734)	0.044(0.017–0.066)	< 0.001

△C-statistics indicated the difference in C-statistic between C-ACS models and models additionally including leukocytes, neutrophils, lymphocytes, monocytes, eosinophils, basophils, NMLR, NLR, MLR, or ELR

*C-ACS* Canada Acute Coronary Syndrome Risk Score, *NMLR* neutrophil-plus-monocyte-to-lymphocyte ratio, *NLR* neutrophil-to-lymphocyte ratio, *MLR* monocyte-to-lymphocyte ratio, *ELR* eosinophil-to-leukocyte ratio

## Discussion

The current prospective study investigated the predictive value of leukocyte parameters for cardiovascular mortality in very old patients with AMI. Cox regression analyses revealed that leukocyte subtypes including neutrophils, lymphocytes, eosinophils, basophils, NMLR, NLR, MLR and ELR were independently associated with cardiovascular mortality in very old patients. However, of all leukocyte parameters, NMLR and NLR were the better predictors of mortality. Moreover, adding NMLR and NLR to both original model and C-ACS model significantly improved the model efficiency. Thus, the present study provided novel information regarding the better performance of NMLR and NLR in predicting cardiovascular mortality in very old patients with AMI.

Previous studies suggested that older patients had not only more severe clinical manifestations but also worse outcomes than younger adult when they are suffered from AMI [23]. In a study of AMI patients, Marrugat, et al. [3] found that the MI mortality, incidence, and case-fatality dramatically increased among patients older than 64 years. Besides, the mortality rate of patients aged 75 to 84 years and 85 to 94 years was higher than that in the 34–64 years age group. They concluded that AMI had a greater impact in the elderly than that in those younger than 65 years old. Similar findings were reported in other studies [24]. Moreover, the problems of the elderly were attracting increasing attention not only in Western countries, but also in Asia. For elderly patients with MI, early risk assessment may help improve the clinical outcome. Thus, it is important to find a simple marker to identify the high-risk elderly population after MI.

Inflammation plays an important role in the occurrence and development of atherosclerotic cardiovascular diseases. Although previous studies have demonstrated that CRP, interleukin-6 and tumor necrosis factor-α were typical biomarkers of inflammation, measuring those markers was expensive and time consuming. Circulating leukocyte is a simple and inexpensive marker from the regular laboratory measurements. A large number of epidemiologic studies have indicated the positive relationship between leukocyte counts and risk of CAD [8–10]. In previous studies, leukocytes were also associated with the severity of CAD [11, 12]. Moreover, accumulating data indicated that leukocytes were predictive of mortality in patients with stable CAD, ACS and AMI [14–18].

However, few studies evaluated which leukocyte subpopulation was the most valuable one in predicting mortality in AMI patients. In 1037 patients with AMI, Dragu et al. [19] found that elevated neutrophil count was most significantly associated with mortality. In another study by Azab et al. [25], it was suggested that NLR was the best predictor of mortality in 1345 patients with non-ST-segment elevation MI. Yet a study by Shiyovich et al. [20] showed that lymphocytes had the strongest association with long-term risk of mortality in patients with AMI. However, these studies did not include all the leukocyte subtypes. Thus, it is still unclear which leukocyte parameter is the best for predicting mortality. In addition, data on the predictive utility of leukocyte and its subpopulations for mortality in very old patients with AMI is not currently available. Also, no study investigated the relation of NMLR and clinical outcome in very old patients with AMI. In our current study, in elderly patients with AMI, we found that most of leukocytes parameters including neutrophils, lymphocytes, eosinophils, basophils, NMLR, NLR, MLR and ELR were significantly and independently associated with mortality after adjustment for confounding variables, among which NMLR and NLR had better predictive performance than the other leukocytes parameters. Thus, the present study firstly reported that NMLR and NLR appeared to be more effective than other leukocytes parameters in predicting mortality in patients over 80 years with AMI.

The use of risk score models for indentifying high-risk patients in AMI is considered as a common method in current clinical guidelines [26, 27]. As one of the risk score models, the C-ACS was a simple model which was previously reported to predict both short- and long-term mortality of patients with ACS [21]. In accordance with previous study [21], our study showed that the C-ACS could independently predict mortality in elderly patients with AMI. As shown in our study, adding NMLR and NLR into the C-ACS model significantly improved the predictive efficiency in mortality. More importantly, adding NMLR and NLR to traditional risk model including age, male gender, BMI, family history of CAD, smoking, hypertension, DM, HDL-C, non-HDL-C, hsCRP, creatinine, LVEF, troponin I, use of statin, ACE-I/ARB, and PCI also significantly improved the model efficiency. Thus, it is necessary to take NMLR and NLR into consideration in the risk stratification for elderly patients with AMI.

As is the main findings of our study, both NMLR and NLR were independent markers and more powerful than other leukocyte parameters in predicting mortality in very old patients with AMI. However, the underlying mechanisms remained unclear. We speculate that the association between NMLR and NLR with mortality may due to their inflammatory and thrombotic activity. In fact, neutrophils could infiltrate vascular wall and result in plaque instability by secretion of superoxide radicals, cytokines, and a variety of proteolytic enzymes [28]. In addition, inflammatory monocytes, which are the earliest responders in AMI, contain many inflammatory cytokines including proteases. These inflammatory cytokines can cause necrosis of myocardium. Lymphocytes, on the other side, could induce the expression of interleukin-10 and promote tissue repairment [29]. Nevertheless, during AMI, subjects often present lower plasma lymphocytes count due to elevated cortisol levels, which may be associated with increased risk of death [30, 31]. Based on above mentioned mechanisms, neutrophils and monocytes play a key role in the inflammatory response while the lymphocytes are involved in the regulation of the immune system. Thereby, NMLR and NLR may reflect the balance between two opposite immune pathways and act as a more powerful predictor for mortality than single differential leukocyte count.

There are several limitations in the current study. First, the sample size of the current study is relatively small, and all patients were recruited from single center. Further studies in larger cohort may be in need to reveal our findings. Second, we only measured the baseline level of leukocytes and its subtypes. Multiple measurements over time may provide more valuable information for predicting mortality. Finally, even if the study only enrolled very old population at baseline, variation for age composition of the study population may affect our findings. Additional analysis of the relationship between white blood cells and outcome for different age subgroups in a larger cohort may also be in need.

## Conclusion

Our data firstly indicated the multiple leukocyte subtypes were independent markers for the mortality in very old patients with AMI, while NMLR and NLR appeared to be more effective, suggesting the necessity to pay more attention to the levels of NMLR and NLR in such patients. In summary, increased NMLR and NLR may serve as simple indicators to identify high-risk patients and help determine appropriate treatment strategies.

## Supplementary information

**Additional file 1: Table S1.** Unadjusted cox proportional hazards regression analysis of cardiovascular mortality.

## Data Availability

The technical appendix, statistical code and data set are available from the corresponding author.

## Abbreviations

CAD: Coronary artery disease
AMI: Acute myocardial infarction
NMLR: Neutrophil-plus-monocyte-to-lymphocyte
NLR: Neutrophil-to-lymphocyte ratio
MLR: Monocyte-to-lymphocyte ratio
ELR: Eosinophil-to-leukocyte ratio
ACS: Acute coronary syndrome
TC: Total cholesterol
DM: Diabetes mellitus
TG: Triglyceride
FPG: Fasting plasma glucose
LDL-C: Low-density lipoprotein cholesterol
HDL-C: High-density lipoprotein cholesterol
hsCRP: High sensitivity C-reactive protein
LVEF: Left ventricular ejection fraction
