# Supplementary material for: Differential leukocyte counts and cardiovascular mortality in very old patients with acute myocardial infarction: a Chinese cohort study
Source: BMC Cardiovasc Disord. 2020 Oct 28;20:465. doi: 10.1186/s12872-020-01743-3 (PMC7594328; doi:10.1186/s12872-020-01743-3)
Supplement: Supplementary file 1 — Additional file 1: Table S1. Unadjusted cox proportional hazards regression analysis of cardiovascular mortality. [file 12872_2020_1743_MOESM1_ESM.docx]

**Additional file Table S1 Unadjusted cox proportional hazards regression analysis of cardiovascular mortality**

| **Variables** | **HR (95%CI)** |  | **P value** |  |
| --- | --- | --- | --- | --- |
| Male | 1.01(0.73-1.39) |  | 0.966 |  |
| Age | 1.09(1.03-1.15) |  | 0.002 |  |
| BMI | 0.94(0.89-0.99) |  | 0.020 |  |
| Smoking | 1.14(0.82-1.58) |  | 0.429 |  |
| Family history of CAD | 1.26(0.78-2.04) |  | 0.341 |  |
| Hypertension | 1.27(0.88-1.84) |  | 0.210 |  |
| Diabetes mellitus | 1.53(1.12-2.11) |  | 0.009 |  |
| HDL-C | 0.57(0.31-1.04) |  | 0.067 |  |
| nonHDL-C | 0.90(0.72-1.12) |  | 0.229 |  |
| hsCRP | 1.07(1.04-1.11) |  | <0.001 |  |
| Creatinine | 1.01(1.01-1.02) |  | <0.001 |  |
| LVEF | 0.96(0.95-0.97) |  | <0.001 |  |
| Troponin I | 1.03(1.01-1.04) |  | <0.001 |  |
| Statin | 0.61(0.37-1.00) |  | 0.051 |  |
| ACE-I/ARB | 0.76(0.55-1.05) |  | 0.098 |  |
| PCI | 0.41(0.29-0.60) |  | <0.001 |  |

BMI, body mass index; CAD, coronary artery disease; DM, diabetes mellitus; HDL-C, high-density lipoprotein cholesterol; hsCRP, high sensitivity C-reactive protein; LVEF: left ventricular ejection fraction; ACE-I: angiotensin-converting enzyme inhibitors; ARB: angiotensin receptor blockers; PCI: percutaneous coronary intervention.
